# Collision cross sections obtained with ion mobility mass spectrometry as new descriptor to predict blood-brain barrier permeation by drugs

**DOI:** 10.1038/s41598-019-55856-7

**Published:** 2019-12-16

**Authors:** Armin Sebastian Guntner, Bernhard Thalhamer, Christian Klampfl, Wolfgang Buchberger

**Affiliations:** Johannes Kepler University Linz, Institute for Analytical Chemistry, Linz, 4040 Austria

**Keywords:** Drug development, Preclinical research

## Abstract

Evaluating the ability of a drug to permeate the blood-brain barrier is not a trivial task due to the structural complexity of the central nervous system. Nevertheless, it is of immense importance to identify related properties of the drugs either to be able to produce a desired effect in the brain or to avoid unwanted side effects there. In the past, multiple methods have been used for that purpose. However, these are sometimes methodologically problematic and do not claim universal validity. Therefore, additional new methods for judging blood-brain barrier penetration by drugs are advantageous. Accordingly, within the scope of this study, we tried to introduce a new structure-derived parameter to predict the blood-brain barrier permeation of small molecules based on ion mobility mass spectrometry experiments – the collision cross section, as an illustration of the branching and the molecular volume of a molecule. In detail, we used ion mobility quadrupole time-of-flight mass spectrometric data of 46 pharmacologically active small-molecules as well as literature-derived permeability and lipophilicity data to set up our model. For the first time we were able to show a strong correlation between the brain penetration of pharmacologically active ingredients and their mass spectrometric collision cross sections.

## Introduction

The determination of a drug’s pharmacokinetic profile especially in terms of its ability to permeate bodily membranes such as the blood-brain barrier (BBB) is of utmost importance not only in daily clinical application but also in the early stages of drug discovery. Drugs targeting the central nervous system (CNS) need to penetrate this barrier in order to produce a desired outcome. On the other hand, peripherally acting substances should not permeate the BBB to avoid possible unwanted side effects. As the development of new pharmaceuticals includes tremendous expenses for the pharmaceutical industry, predicting the expectable permeation properties of a substance prior to further costly studies is a significant step in improving the cost-effective discovery of new potential CNS drugs. It has been shown that physicochemical properties influencing the pharmacokinetics (PK) are in general of equal importance as the pharmacodynamic (PD) profile for a drug to become market-ready, as many of the substances with promising activity developed in the past failed in terms of permeability. Consequently, developers aim to increase the percentage of substances passing the preclinical studies, but nonetheless have to accept a high rate of failure based on the structural complexity of the brain and its protective layer the BBB. The BBB itself is built of endothelial cells that infold the blood vessels within the CNS, and comprise intercellular tight junctions as well as uptake and efflux transport systems that in total regulate the permeation of exogenous substances. However, the vast majority of small molecule drugs migrates into the brain driven by passive diffusion depending on their physicochemical properties^1–10^.

Different assays based on *in vitro*, *in vivo* and *in silico* experiments have been developed earlier in order to obtain information on the likelihood of a drug permeating into the CNS. Unfortunately, they often show conflicting results, proving the need of new methodologies. *In vitro* experiments are mainly conducted as a filtering approach where either artificial membranes mimicking biological lipid layers (PAMPA) or endothelial cells are used to separate donor and acceptor compartments. The permeation into the latter is then assessed on the basis of LC-UV or LC-MS/MS analysis^2,9,11,12^.

*In vivo* experiments on rodents have been intensively used in the past to assess the total mammalian brain level of CNS drugs. However, conclusions drawn from such studies might be misleading as for example the efflux transporter breast cancer resistance protein (BCRP) is around twice as pronounced in humans as in rodents. Still, mammalian pharmacokinetic data from larger animals or humans are in general barely available because of the methodological complexity. One possibility to acquire respective data is to use cerebrospinal fluid as a surrogate for CNS exposure, taken either by lumbar puncture or by ventricular drainage using an Ommaya reservoir. Another possibility to assess human *in vivo* cerebral PK data is the implementation of a cerebral microdialysis catheter. Although this technique shows some significant drawbacks such as the severely invasive character and the need for elaborate calibration to determine the recovery of the system, it does provide a tremendous advantage. Only with this method it is possible to directly sample the unbound fraction of an active ingredient at the target site, representing the level that is actually having pharmacological effects^3,6,9,13–18^.

**Figure 1:**
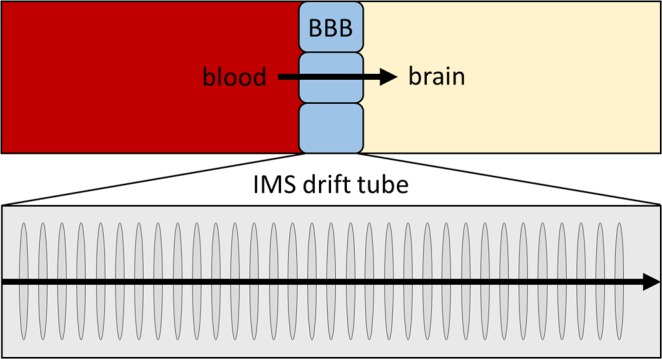
Graphical representation how to assess the blood-brain barrier penetration of small-molecule pharmaceuticals using a drift tube ion mobility mass spectrometer. The upper panel of the illustration represents the blood-brain barrier between blood and brain. The lower panel of the figure represents the drift tube of an IMS-QTOF MS used to evaluate BBB penetration properties of molecules within the framework of the present study. In both panels, the arrows represent the passage of compounds through an impeding barrier.

In addition to *in vivo* and *in vitro* experiments that provide most valuable PK data but may be ethically problematic, *in silico* methods based on physicochemical parameters are used to estimate the permeation of pharmaceuticals through the BBB. Especially the “Lipinksi rule of 5”^19^ provides a generally accepted proposal for the estimation of the permeation, but was extended in recent years. Pajouhesh and Lenz^10^ have summarized in detail parameters that are used to estimate cerebral PK. Besides lipophilicity, molecular weight, the number of hydrogen bonding acceptor/donors, polar surface area and pKa, also the molecular volume and the flexibility are listed as crucial factors^11^. Furthermore Gerebtzoff and Seelig^20^ have shown a correlation of cerebral PK data with calculated molecular cross-sectional areas of drugs orientating themselves in the amphiphilic environment of the BBB. Considering the latter fact, as well as molecular volume and flexibility as suitable parameters for assessing the penetration of the BBB by a pharmaceutical, it becomes reasonable to use an analytical technique that exactly represents them. In this context, Fig. 1 shows the approach suggested in the present study to assess BBB penetration by active pharmaceutical agents based on drift tube ion mobility quadrupole time-of-flight mass spectrometer (IMS-QTOF MS) experiments.

Ion mobility mass spectrometry is a technique, where analyte molecules are ionized by means of electrospray ionization and are separated in a first step according to their drift times within a drift tube, and in a second step according to their mass over charge ratio in a hyphenated mass spectrometer. Drift time (DT) separation is achieved by moving ions within an electrical field in a tube filled with an inert drift gas such as nitrogen. As a related molecular characteristic the collision cross section (CCS) is therewith obtained, reflecting the two dimensional projection of the sphere that is formed by the randomly rotating ionized molecule in the gas phase. Mathematically this can be described by the Mason-Schamp equation^21–23^:

1 $${}^{{\rm{DT}}}{\rm{C}}{{\rm{CS}}}_{{{\rm{N}}}_{2}}=\frac{{(18\pi )}^{\frac{1}{2}}}{16}\frac{ze}{{({k}_{b}T)}^{\frac{1}{2}}}{(\frac{1}{{m}_{i}}+\frac{1}{{m}_{B}})}^{\frac{1}{2}}\frac{{t}_{A}E}{L}\frac{760}{P}\frac{T}{273.15}\frac{1}{N}$$

In this case, the given variables describe:

*z* ion charge number, *e* elementary charge, *k*_*b*_ Boltzmann constant, *T* temperature, *m*_*i*_ ion mass, *m*_*B*_ neutral gas mass, *t*_*A*_ arrival time, *E* electrical field, *L* length of the drift tube, *P* drift tube pressure, and *N* neutral gas number density^21^.

Further, the total arrival time at the detector in an ion mobility experiment needs to be considered as the sum of the real drift time *t*_*D*_ within the drift tube and the dead time *t*_*fix*_:

2 $${t}_{A}={t}_{{\rm{D}}}+{t}_{fix}$$

Consequently, a calibration based on standard substances is necessary to transform an arrival time into a ${}^{{\rm{DT}}}{\rm{C}}{{\rm{CS}}}_{{{\rm{N}}}_{2}}$ value. For that purpose in a single-field experimental approach, under constant conditions, most parameters of Eq. (1) can be combined (*β*) and following Eq. (3) can be used to calibrate the system and eventually calculate ${}^{{\rm{DT}}}{\rm{C}}{{\rm{CS}}}_{{{\rm{N}}}_{2}}$ values^21^:

3 $${t}_{A}=\frac{\beta }{z}\sqrt{\frac{{m}_{i}}{{m}_{i}+{m}_{B}}}{}^{{\rm{DT}}}{\rm{C}}{{\rm{CS}}}_{{{\rm{N}}}_{2}}+{t}_{{\rm{fix}}}$$

## Results

**Figure 2:**
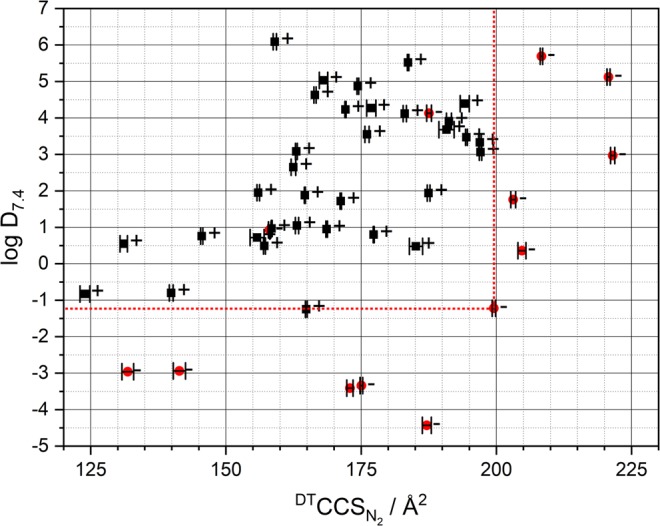
Depiction of the calculated log D_7.4_ data and the measured nitrogen drift-time CCS values. Red circles indicate here substances without sufficient permeation of the BBB as known from literature. Black boxes represent molecules that permeate into the CNS. Error bars represent standard deviation of employed six measurements. The dashed red lines are the limits as given by our study for substances to penetrate the BBB.

As the determination of pharmacokinetic properties of drugs regarding their ability to permeate the BBB is relevant in early stages of drug discovery but also in further clinical applications, the introduction of a new structure derived molecular parameter to evaluate the penetration properties of a substance is favourable. We herein present the results of our study correlating drift time ${}^{{\rm{DT}}}{\rm{C}}{{\rm{CS}}}_{{{\rm{N}}}_{2}}$ values supplemented by lipophilicity data with BBB permeation of an active ingredient of a pharmaceutical formulation. In this context, Table 1 and Fig. 2 provide the respective data.

**Table 1:** Overview of the data obtained within the present study.

Substance	${}^{{\rm{DT}}}{\rm{C}}{{\rm{CS}}}_{{{\rm{N}}}_{2}}$/Å^2^	Ion Polarity	Species	Δ${}^{{\rm{DT}}}{\rm{C}}{{\rm{CS}}}_{{{\rm{N}}}_{2}}$/Å^2^	Mass Accuracy, Δppm	BBB	pKa	log P	log D_7.4_
Ampicillin	187.14	+	(M + H)^+^	−12.96	−5.03	−	3.17	−0.2	−4.43
Aspirin	131.84	−	(M − H)^−^	7.12	5.59	−	3.26	1.18	−2.96
Cetirizine	199.54	+	(M + H)^+^	11.39	−0.90	−	3.08	3.11	−1.22
Cimetidine	158.09	+	(M + H)^+^	20.58	2.57	−	−	0.93	0.93
Domperidon	203.12	+	(M + H)^+^	25.31	−4.03	−	8.39	1.8	1.76
Fexofenadine	221.52	+	(M + H)^+^	18.38	−12.91	−	4.34	6.03	2.97
Fluvastatin	204.72	+	(M + H)^+^	−5.49	−1.05	−	4.35	3.41	0.36
Furosemid	172.93	−	(M − H)^−^	4.27	0.67	−	3.25	0.74	−3.41
Loperamid	220.75	+	(M + H)^+^	6.33	−6.32	−	8.59	5.15	5.12
Loratadine	187.54	+	(M + H)^+^	22.46	−16.96	−	−	4.13	4.13
Mannitol	135.57	+	(M + H)^+^	−12.45	2.64	−	15.04	−2.94	−2.94
Penicillin G	175.05	+	(M + H)^+^	8.80	8.95	−	3.22	0.84	−3.34
Verapamil	208.32	+	(M + H)^+^	27.51	0.66	−	9.47	5.69	5.69
Alprazolam	172.11	+	(M + H)^+^	19.66	−7.33	+	−	4.23	4.23
Amitriptylin	166.47	+	(M + H)^+^	13.37	−0.24	+	9.9	4.63	4.63
Atenolol	157.13	+	(M + H)^+^	20.25	−2.17	+	9.23	0.5	0.49
Bisoprolol	187.47	+	(M + H)^+^	14.21	−3.27	+	14.05	1.94	1.94
Caffeine	139.89	+	(M + H)^+^	5.62	2.39	+	10.4	−0.8	−0.80
Clomipramin	174.41	+	(M + H)^+^	0.59	9.94	+	9.76	4.87	4.87
Clonidine	145.54	+	(M + H)^+^	11.21	7.25	+	8.16	0.83	0.76
Dehydrocotisol	185.14	+	(M + H)^+^	−5.83	10.29	+	14.71	0.48	0.48
Desloratadine	176.09	+	(M + H)^+^	2.35	−3.59	+	9.22	3.56	3.55
Diazepam	164.86	+	(M + H)^+^	7.12	5.38	+	3.17	2.98	−1.25
Dronabinol	183.68	+	(M + H)^+^	−4.88	−1.00	+	9.32	5.53	5.52
Estradiol	159.04	+	(M + H)^+^	3.98	18.55	+	9.58	3.91	6.09
Fluoxetine	176.88	+	(M + H)^+^	0.56	−1.24	+	9.78	4.27	4.27
Haloperidol	194.47	+	(M + H)^+^	3.41	−3.54	+	8.66	3.49	3.47
Hydroxyzine	196.95	+	(M + H)^+^	7.98	2.62	+	14.48	3.33	3.33
Ibuprofen	155.77	−	(M − H)^−^	0.82	−3.01	+	4.37	3.75	0.72
Lidocain	155.99	+	(M + H)^+^	1.42	−1.13	+	7.12	2.41	1.95
Lovastatin	190.78	+	(M + H)^+^	−0.72	0.33	+	13.17	3.68	3.68
Mefloquine	183.06	+	(M + H)^+^	−1.42	−14.46	+	13.14	4.12	4.12
Metoprolol	171.24	+	(M + H)^+^	19.25	2.18	+	9.68	1.72	1.72
Morphine	164.60	+	(M + H)^+^	3.85	−12.23	+	14	1.88	1.88
Naltrexon	177.31	+	(M + H)^+^	−4.61	0.05	+	8.83	0.82	0.80
Nebivolol	197.07	+	(M + H)^+^	3.29	2.34	+	7.87	3.19	3.06
Oxacepam	163.03	+	(M + H)^+^	2.18	−3.08	+	11.65	3.08	3.08
Paracetamol	131.09	+	(M + H)^+^	−3.99	14.69	+	9.76	0.55	0.55
Piracetam	123.91	+	(M + H)^+^	3.88	−15.61	+	12.88	−0.83	−0.83
Propanolol	162.48	+	(M + H)^+^	3.21	−17.87	+	14.04	2.65	2.65
Quetiapine	191.23	+	(M + H)^+^	4.06	−0.13	+	14.48	3.91	3.91
Ranitidine	168.63	+	(M + H)^+^	49.85	−9.10	+	8.57	0.98	0.95
Salbutamol	158.46	+	(M + H)^+^	−3.90	−2.91	+	9.42	0.97	0.97
Sertralin	168.04	+	(M + H)^+^	7.05	3.70	+	9.58	5.03	5.03
Simvastatin	194.15	+	(M + H)^+^	6.26	4.05	+	13.17	4.39	4.39
Sumatriptan	163.14	+	(M + H)^+^	17.07	−14.35	+	9.94	1.05	1.05

${}^{{\rm{DT}}}{\rm{C}}{{\rm{CS}}}_{{{\rm{N}}}_{2}}$ values of the examined compounds respectively the corresponding ionized species are shown. ∆${}^{{\rm{DT}}}{\rm{C}}{{\rm{CS}}}_{{{\rm{N}}}_{2}}$ and ∆ppm represent the differences of measurement data and theoretical values, obtained from literature and with the software stated in the Materials and Methods section of the text respectively.

**Figure 3:**
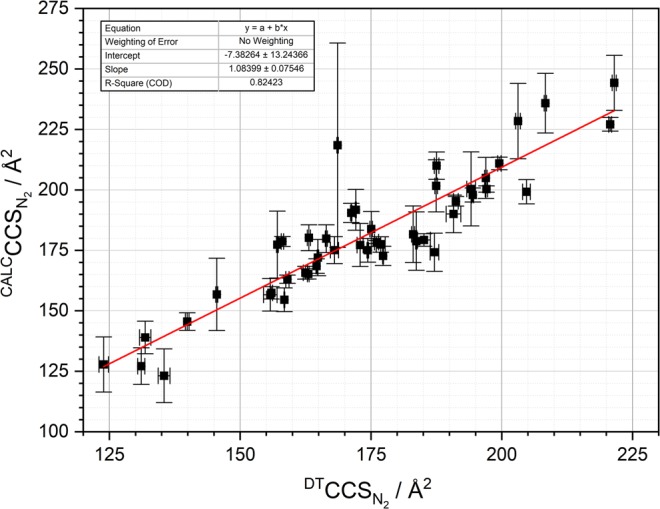
Depiction of measured nitrogen drift-time CCS values versus mean calculated CCS values using IMoS, PSA, and MetCCS software (both given in Ångstroms squared). The linear regression is given in red.

As described in further detail in Table 1 the listed substances were analysed by the means of IMS-QTOF MS in both polarities depending on the nature of the analytes. Multiply charged ions were neglected in the present study and mostly proton adducts were considered. ∆${}^{{\rm{DT}}}{\rm{C}}{{\rm{CS}}}_{{{\rm{N}}}_{2}}$ was computed as the difference of measured ${}^{{\rm{DT}}}{\rm{C}}{{\rm{CS}}}_{{{\rm{N}}}_{2}}$ values and calculated average of CCS values of IMoS^24^, PSA^25–28^ and MetCCS^29,30^. In this context, Fig. 3 provides visual output of the high correlation between measured and predicted CCS values, especially when used in a combined manner. Measured ${}^{{\rm{DT}}}{\rm{C}}{{\rm{CS}}}_{{{\rm{N}}}_{2}}$ values were highly reproducible, as described in further detail below. In Table 1 the mass accuracy Δppm represents the difference between measured m/z values and theoretical ones. In fact, 91% of the mass measurements were better than 20 ppm mass error. Deviations are hereby based on the fact that within IM measurements reference mass correction of QTOF measurements is not possible. As described below Chemdraw derived pKa and log P data was used to calculate log D_7.4_, according to Eq. (4) ^20,31,32^.

In the literature^33–35^ it has been stated that different adducts originating from the same analyte molecule (e.g. charged solvent-analyte-clusters) may be observed in ion mobility analysis. This however has to be considered as one possible source of an error in reporting ${}^{{\rm{DT}}}{\rm{C}}{{\rm{CS}}}_{{{\rm{N}}}_{2}}$ values as differing adducts have different mobilities in the drift tube and may be stable due to the increased pressure there. They however fragment eventually in the high-vacuum of the hyphenated mass spectrometer and therefore lead to signals at the same m/z but with varying corresponding mobility signals. One possibility to encounter this phenomenon is to check for the lowest drift time signal of a given m/z as this is most probably related to the (M + H)^+^ ion in positive polarity. We however tried to ensure the accuracy of our reported ${}^{{\rm{DT}}}{\rm{C}}{{\rm{CS}}}_{{{\rm{N}}}_{2}}$ values by variation of the instrumental conditions prior to IMS-Q-TOF MS to avoid the misinterpretation of ion mobility signals. One-way analysis of variance proved a non-significant influence of the solvent system (methanol, acetonitrile and isopropanol) used. We were able to determine here a maximum relative standard deviation (calculated taking into account all six measurements in the three solvents) of the specific ${}^{{\rm{DT}}}{\rm{C}}{{\rm{CS}}}_{{{\rm{N}}}_{2}}$ values for all analytes below 0.84%. The mean RSD was calculated to be 0.27%. Within intraday experiments, the precision of the ion mobility measurements, represented as RSD, was determined to be 0.19%. Furthermore, we tried to determine the precision within ten measurements for an analyte with low matrix induced suppression. Here we were able to show that the determined ${}^{{\rm{DT}}}{\rm{C}}{{\rm{CS}}}_{{{\rm{N}}}_{2}}$ values of naltrexone had a very high precision with 0.02% RSD.

As to be seen in Fig. 2, plotting the investigated substances according to their log D_7.4_ and their ${}^{{\rm{DT}}}{\rm{C}}{{\rm{CS}}}_{{{\rm{N}}}_{2}}$ values a distinct correlation to their ability to cross the BBB is visible. Due to the general lack of information on the numeral probability of BBB penetration only binary statements were used here. In Fig. 2, the dashed red line represents the limits in terms of lipophilicity and collision cross section for a substance to enter the central nervous system. As long as $\log \,{{\rm{D}}}_{7.4}\ge -\,1.2$ and ${}^{{\rm{D}}{\rm{T}}}{\rm{C}}{{\rm{C}}{\rm{S}}}_{{{\rm{N}}}_{2}}\le 199.5\,{\AA }^{2}$ drugs seem to penetrate the blood-brain barrier, and enter the central nervous system based on passive diffusion. These results are in strong correlation to the data shown by Gerebtzoff and Seelig^20^, who calculated the cross-sectional areas of molecules perpendicular to their amphiphilicity axis eventually linking them to penetration properties. We in contrast measured the two-dimensional projection of the forming sphere of a freely rotating molecule in the gas phase and correlated the respective data with known permeabilities. Two substances (Cimetidine (log D_7.4_ = 0.93 and ${}^{{\rm{D}}{\rm{T}}}{\rm{C}}{{\rm{C}}{\rm{S}}}_{{{\rm{N}}}_{2}}$ $=158.09\,{\AA }^{2}$) and Loratadine (log D_7.4_ = 4.13 and ${}^{{\rm{D}}{\rm{T}}}{\rm{C}}{{\rm{C}}{\rm{S}}}_{{{\rm{N}}}_{2}}=187.54\,{\AA }^{2}$)) are not conforming to the overall result as their presence in the CNS is system is known to be limited. This however can be related to their substrate character for P-gp efflux pumps leading to a reduced CNS exposition^36–38^.

In addition to the instrumental analysis of ion mobilities, a software-based prediction of CCS data was performed using IMoS, PSA and MetCCS in a combined manner. In this context, Fig. 3 shows the correlation of predicted and measured collision cross sections. Considering, that all software-based prognosis was in satisfactory correlation (R^2^ > 0.82) with measured collision cross sections and was achieved with free software, our model seems to be applicable also to preliminary pharmacokinetic investigations without the access to costly, but high-performing ion mobility instruments.

## Discussion

For the first time, in this work, we were able to correlate the cerebral pharmacokinetics of active substances with their collision cross sections in the gas phase. In addition to the instrumental ascertainment of this parameter, we were also able to use software-based prediction of CCS values, enabling a preliminary forecast without access to costly ion mobility instruments. The accurate calculation of CCS values however is apart from the performance of the employed prediction tools strongly depended on the accuracy of the input structures and the level of theory used. Within the framework of this research only low-level computational theory was employed to show that first statements can be made with simple means supporting the instrumental determination. However, it has to be stated that our instrumental procedure allows a high-throughput ascertainment of the likelihood of substances penetrating the BBB and is - considering high accuracy and reproducibility of the measurements – in general superior to *in silico* determinations. Moreover, it should be noted that the increasing availability of online CCS databases allows easy access to corresponding information proving additionally the advantage of CCS values as a valid descriptor to assess the penetration of the blood-brain barrier by a molecule.

However, it has to be stated that the measurement of CCS values cannot fully replace *in vivo* studies in rodents or larger mammalians such as humans to assess cerebral pharmacokinetics. This is due to the circumstance that besides structure and non-structure derived factors, the substrate character of small-molecules to efflux-pumps significantly influences the CNS exposure. Consequently, the cerebral pharmacokinetic profile of a drug might be changed completely contrary to the physicochemical parameters, which, however, does not necessarily diminish their importance, especially when considering the early stages of pharmaceutical development.

Although, we employed a binary (yes/no) consideration, we were still able to introduce a new parameter that allows us to quantitatively measure the molecular volume and the molecular branching of an active substance. Both have been described as relevant to permeability in the past, but have not been investigated adequately to date. In fact, collision cross sections appear to be an additional molecular descriptor besides lipophilicity, molecular weight and others to pre-estimate cerebral pharmacokinetic properties of active substances. Especially in the threshold region CCS values are complementing molecular mass and other parameters as descriptor for BBB penetration of compounds. Various reported limits for sufficient permeation may be ambiguous so that CCS values may avoid false positive/negative results. Further, it is generally beneficial to determine numerous structure and non-structure related parameters for an expanded characterization. Consequently, a combined approach is most appropriate to prevent incorrect conclusions, as actual permeabilities may be in accordance with certain reported molecular descriptor limits for sufficient permeation, but may not meet others. In other words, the employment of synergistic descriptors allows a profound assessment of the likelihood of BBB penetration by compounds at an early stage in drug discovery. In summary, CCS values present a new possibility to predict in advance the applicability of a compound for the treatment of diseases in the central nervous system, or to determine at an early stage a potential unwanted adverse effect there. Especially because the development of pharmaceuticals is a time-consuming and costly procedure, the approach we present here, provides, in our eyes, a valuable, new, fast, and easy option to assess the cerebral pharmacokinetic behavior of a drug.

## Materials and Methods

### Instrumentation and chemicals

All analyses in the present study were performed with a 1200 Series HPLC instrument operated in the flow-injection mode without a separation column hyphenated to a 6560 Ion-Mobility Quadrupole Time-of-Flight MS (IMS-Q-TOF MS), both from Agilent Technologies (Santa Clara, California).

All used solvents were HPLC grade and were purchased from VWR International GmbH (Darmstadt, Germany). Water was used in Millipore quality obtained from a Millipore purification system (Molsheim, France). Formic acid (≥96%) was purchased from Sigma-Aldrich Handels GmbH (Vienna, Austria).

In the context of this work 46 different active ingredients of pharmaceutic formulations, resembling different drug classes, were tested. The selection of substances was based on the accessibility of BBB permeation data of the respective compounds and the commercial availability of appropriate formulations. As numeral BBB penetration probability is generally unavailable in literature, only binary statements were used. All pharmaceuticals in the present study were obtained from a local pharmacy under presentation of a corresponding license. The pure substances listed below were purchased from Sigma-Aldrich Handels GmbH (Vienna, Austria). In this context, Table 2 provides an overview of the substances under investigation and their ability to penetrate the BBB as given in literature.

**Table 2:** Overview of the tested substances including information on BBB permeability and manufacturer.

Active substance	BBB Permeability		Formulation	Manufacturer
Ampicillin	−	^20^	Unasyan	Pfizer
Aspirin	−	^39^	Aspro	MCM Klosterfrau
Cetirizine	−	^20^	Cetiristad	Stada
Cimetidine	−	^20^	Ulcostad	Stada
Domperidone	−	^20^	Motilium	Janssen
Fexofenadine	−	^20^	Allegra	Sanofi
Fluvastatin	−	^40^	Fluvastatin	Stada
Furosemide	−	^20^	Furohexal	Hexal
Loperamide	−	^20^	Enterobene	Ratiopharm
Loratadine	−	^20^	Loratadin Alternova	Krka
Mannitol	−	^20^	Mannitol	BDA microanalytical reagents
Penicillin G	−	^20^	Penicillin-G Natrium	Sandoz
Verapamil	−	^20^	Verabene	Ratiopharm
Alprazolam	+	^20^	Alprazolam	Ratiopharm
Amitriptyline	+	^20^	Saroten	Lundbeck
Atenolol	+	^41^	Atenolol	1A Pharma
Bisoprolol	+	^42^	Bisoprolol	Sandoz
Caffeine	+	^43^	Coffekapton	Strallhofer Pharma GmbH
Clomipramine	+	^20^	Anafranil	Novartis
Clonidine	+	^20^	Catapresan	Boehringer Ingelheim
Dehydrocortisol	+	^44^	Prednisolon	Takeda
Desloratadine	+	^45^	Desloratadine	Genericon Pharma
Diazepam	+	^20^	Gewalcam	Takeda
Dronabinol	+	^46^	Dronabinol	Bionorica
Estradiol	+	^47^	Estradiol	Sigma Aldrich
Fluoxetine	+	^20^	Mutan	Gerot Lannach
Haloperidol	+	^20^	Haldol	Janssen
Hydroxyzine	+	^48^	Atarax	UCB Pharma
Ibuprofen	+	^49^	Ibuprofen	Genericon Pharma
Lidocaine	+	^20^	Lidocain	Apotheke
Lovastatine	+	^40^	Lovastatine	KRKA
Mefloquine	+	^50^	Lariam	Roche
Metoprolol	+	^20^	Metoprolol Genericon	Genericon Pharma
Morphine	+	^20^	Morapid	Mundi Pharma
Naltrexone	+	^20^	Dependex	Amomed
Nebivolol	+	^51^	Nomexor	A. Menarini Pharma GmbH
Oxacepam	+	^20^	Praxiten	Meda
Paracetamol	+	^52^	Paracetamol	Genericon Pharma
Piracetam	+	^20^	Cerebryl	Kwizda Pharma
Propanolol	+	^53^	Inderal	Astra Zeneca
Quetiapine	+	^54^	Seroquel	Astra Zeneca
Ranitidine	+	^55^	Ranic	Hexal
Salbutamol	+	^20^	Sultanol	GSK Pharma GmbH
Sertraline	+	^56^	Sertraline	1 A Pharma
Simvastatin	+	^57^	Simvastatin	+pharma
Sumatriptan	+	^20^	Sumatriptan	1 A Pharma

−/+Represent here the substance’s likelihood of BBB permeation.

### Sample preparation

Prior to IMS-Q-TOF MS analysis, all pharmaceuticals listed in Table 2 were dissolved in methanol and stepwise diluted to 1 mg/L. Methanolic stock solutions were prepared in 50 mL volumetric flasks at room temperature and homogenization was achieved using ultrasound. Insoluble components were filtered off using appropriate syringe filters. Injection solutions contained only one analyte at a time.

Ampicillin was formed *in situ* using hydrolysis of its prodrug sultampicillin.

### IMS-Q-TOF MS conditions

The IMS-Q-TOF MS was operated in both polarities depending on the analyte, within a mass range of 100–1000 m/z. A frame rate of 1 frame/s, an ion mobility transient rate of 16 transients/frame, a trap fill time of 20 ms and a trap release time of 150 µs were set. All ion mobility experiments were conducted with nitrogen as drift gas. Electrospray ionization conditions included a nebulizer pressure of 3.45 bar, a drying gas temperature of 300 °C at a flow rate of 10 L/min and a sheath gas temperature of 350 °C at a flow rate of 11 L/min. The capillary voltage was set to 3.5 kV and the nozzle voltage to 1 kV.

Prior to analysis, a transmission tune of the 6560 IMS-Q-TOF MS was performed to assure accuracy and performance. The tune was done in an extended dynamic range 2 GHz high-resolution mode in positive and in negative ion polarity. The ion mobility resolution was well above 50 in positive polarity and around 40 in the negative mode for an acquisition range between 100–1000 m/z.

As is known proton, sodium, potassium, ammonium adducts may be formed due to positive electrospray ionization, while chloride and bromide adducts may form or proton loss may occur due to negative electrospray ionization. In fact, different ion species tend to have quite different collision cross sections, causing a potential source of error. For that reason, in all analyses of the present study, the ion species reflecting most accurately solely the molecule itself was chosen to evaluate ${}^{{\rm{DT}}}{\rm{C}}{{\rm{CS}}}_{{{\rm{N}}}_{2}}$ values. In positive polarity of the mass spectrometer consequently the (M + H)^+^ ions were analysed, in negative mode (M − H)^−^ ions respectively.

The analytes of the present study were introduced into the system in a flow-injection approach using a hyphenated HPLC. The corresponding instrumental conditions included thereby the application of an isocratic mobile phase consisting of either methanol, acetonitrile, or isopropanol in combination with 0.1% v/v aqueous formic acid in a ratio of 70/30 in positive ion polarity mode. In negative polarity mode a 30 mM ammonia solution in methanol, acetonitrile, or isopropanol was used. In all cases no analytical column was utilized because of the flow-injection character of all analyses. Mobile phase flow rate was fixed to 0.5 mL/min at 40 °C in all experiments and an injection volume of 10 µL was used. The run time was set to 4 min for each sample and each sample was measured six times (two times for each organic solvent).

### Data processing and software

Data processing was achieved in all cases using MassHunter Workstation Software IM-MS Browser B.08.00 from Agilent Technologies (Santa Clara, California) as well as MassHunter Workstation Software Mass Profiler B.08.00 from Agilent Technologies (Santa Clara, California). The former was used for calibrating ion mobility data (see to Eqs. 3 and 4) and manual data evaluation, the latter for batchwise data evaluation of ion mobility measurements in a feature extraction approach.

Feature extraction parameters of the Mass Profiler software included a minimum ion intensity of 1000 and a mass tolerance of 15 ppm. Q-Score filtering was turned off and the charge state was restricted to one. Compounds that were not found by automated feature extraction were determined manually. In all cases, measured arrival times were transformed into ${}^{{\rm{DT}}}{\rm{C}}{{\rm{CS}}}_{{{\rm{N}}}_{2}}$ values according to a single field calibration approach, as stated above.

In addition, to confirm the accuracy of the measurements, the ${}^{{\rm{DT}}}{\rm{C}}{{\rm{CS}}}_{{{\rm{N}}}_{2}}$ values obtained according to IMS-Q-TOF MS experiments were compared with calculated CCS values based on results from MetCCS webserver^29,30^, PSA webserver 0.5.1^25–28^ and IMoS 1.08 software^24^. Data input for the respective software solutions to calculate CCS values was generated using ChemDraw 17.1.0.105 and Chem3D 17.1.0.105 (Perkin Elmer, Waltham, Massachusetts) as well as OpenBabel 2.3.0 for the conversion of chemical data file formats as needed.

Tables and figures were created using Microsoft Excel 2016 and Microsoft Powerpoint 2016 (Redmond, Washington) as well as OriginLab Corporation OriginPro 2019b (Northampton, Massachusetts).

### Calculation of log D_7.4_

The octanol-water partition coefficient at pH 7.4 log D_7.4_ was calculated according to the following formula^20,31,32^:

4 $$\log \,{{\rm{D}}}_{7.4}=\,\log \,{\rm{P}}-\,\log (1+{10}^{{\rm{pH}}-{\rm{pKa}}})$$

Data for log P and pKa were obtained using ChemDraw 17.1.0.105 (Perkin Elmer, Waltham, Massachusetts) data and are therefore theoretical values rather than empirical values.

## Data Availability

The datasets generated during the current study are available from the corresponding author on reasonable request.
